# What Defines NK Cell Functional Fate: Phenotype or Metabolism?

**DOI:** 10.3389/fimmu.2019.01414

**Published:** 2019-06-19

**Authors:** Sophie M. Poznanski, Ali A. Ashkar

**Affiliations:** Department of Pathology and Molecular Medicine, McMaster Immunology Research Centre, McMaster University, Hamilton, ON, Canada

**Keywords:** NK cell, cell metabolism, phenotype, NK cell subsets, glycolysis, mitochondria, CD56, innate immunity

## Abstract

NK cells are capable of an array of functions that range widely from their classic anti-tumor and anti-viral cytotoxic effector functions, to their critical regulatory roles in controlling inflammatory immune responses and promoting tissue growth. However, the mechanisms that polarize NK cells to these distinct and opposing functions are incompletely understood. NK cell functional subsets are primarily identified and studied based on phenotype, which has served as an accessible means for profiling NK cells and does offer information on NK cell activation state. However, inconsistencies have emerged in using classic phenotypes to inform function, which raise the questions: Can phenotype in fact define NK cell functional fate? What factors do profile and drive NK cell fate? In other immune cells, cell metabolism has been shown to critically determine subset polarization. There is a growing body of evidence that cell metabolism is integral to NK cell effector functions. Glucose-driven glycolysis and oxidative metabolism have been shown to drive classic NK cell anti-tumor and anti-viral effector functions. Recent studies have uncovered a critical role for metabolism in NK cell development, education, and memory generation. In this review, we will draw on the evidence to date to investigate the relationship between NK cell phenotype, metabolism, and functional fate. We explore a paradigm in which the differential activity of metabolic pathways within NK cells produce distinct metabolic fingerprints that comprehensively distinguish and drive the range of NK cell functional abilities. We will discuss future areas of study that are needed to develop and test this paradigm and suggest strategies to efficiently profile NK cells based on metabolism. Given the emerging role of metabolism in driving NK cell fates, profiling and modulating NK cell metabolism holds profound therapeutic potential to tune inflammatory and regulatory NK cell responses to treat disease.

## Introduction

Natural Killer (NK) immune cells are capable of an array of functions that range widely from their classic innate anti-tumor and anti-viral effector functions, to their regulatory roles in modulating other immune cells and promoting tissue growth (1). These distinct functions play critical yet paradoxical roles in a host of diseases. Cytotoxic NK cells are known to have an imperative role in the clearance of virally-infected and malignant cells (2); however, signals in the tumor environment polarize NK cells to regulatory subsets that support tumor progression and suppress other cytotoxic immune cells (3–5). While pathogenic in the context of cancer, regulatory NK cells have critical homeostatic roles in tissue growth and immune tolerance in contexts such as placental development and fetal tolerance in pregnancy (6–8), and regulating fibrosis and immune cells in the liver (9–11). Despite the critical importance of mounting the appropriate NK cell response in different disease contexts, the mechanisms that polarize NK cells to these distinct and opposing functional fates are incompletely understood.

To date, NK cell functional subsets have been primarily identified and studied based on phenotypic markers. In their most classic definition, human NK cells are broadly dichotomized into CD56^bright^CD16^−^ regulatory NK cells with greater cytokine-producing capabilities, and CD56^dim^CD16^+^ anti-tumor/anti-viral NK cells with greater cytotoxic functions (12, 13). Additional receptor families function to tune NK cell activation and effector responses; these include activating Natural Cytotoxicity Receptors (NKp30, NKp44, and NKp46), activating and inhibitory CD94/NKG2 receptors that recognize non-classical MHC, and inhibitory KIR receptors that recognize classical MHC. The differential expression of these receptors is used to further specify NK cell developmental stages, effector subsets, and memory populations (14). Although phenotype is an accessible means by which to profile NK cells and provides information on NK cell activation state, reports are increasingly emerging that highlight discrepancies between NK cell phenotypic classification and their effector functions. These discrepancies present the questions: Can phenotype in fact define NK cell functional fate? What factors do define and drive NK cell fate?

A cell's ability to generate energy through metabolism enables its functional capacities. Indeed, there is a growing body of evidence that cell metabolism is integral to NK cell effector functions. It has been established that glucose-driven glycolysis and oxidative metabolism are required for classic NK cell anti-tumor and anti-viral effector functions (15–18). Recent studies have also emerged that point to critical roles of metabolism in NK cell development, education, and memory responses (19–23). In other immune cell subsets including T cells and macrophages, cell metabolism critically determines subset polarization. Up-regulation of glycolytic metabolism drives the polarization of pro-inflammatory T cells and macrophages (24–27). In contrast, a shift to respiration-derived ATP drives the polarization and functions of regulatory macrophages and T cells (24–26, 28). The generation of memory in T cells is marked by an increase in mitochondrial respiratory capacity, which enables a more rapid and robust secondary immune response (29, 30). Even within a terminally polarized subset, the activation of different metabolic pathways leads to distinct functional outcomes (31). While metabolism has been shown to regulate certain NK cell effector functions, the role of metabolism in broadly determining and defining different NK cell functional fates remains to be fully characterized.

Herein, we explore the relationship between NK cell phenotype, metabolism, and functional fate. Drawing on the evidence to date, we investigate the utility and roles of NK cell phenotype and metabolic activity in identifying and determining NK cell effector fate. We propose that differential activity in NK cell metabolic pathways, but not phenotype, produces distinct and subset-defining fingerprints that comprehensively distinguish and drive the range of possible NK cell functional abilities.

## NK Cell Fate Based on Phenotype: Can We Judge a Book by its Cover?

For decades, NK cell phenotype has been integrally conflated with effector function and used as the principle means of classifying NK cells into distinct subsets. For instance, the stages of NK cell maturation are demarcated by the expression of specific phenotypic markers. Less mature human peripheral blood (pb)NK cells are defined as CD56^bright^CD16^−^ and express CD94 and the NKG2A inhibitory receptor (32, 33). As NK cells mature, they down-regulate CD56 expression and up-regulate CD16, becoming CD56^dim^CD16^+^ (34). These lose expression of NKG2A and acquire expression of KIR inhibitory receptors. A final step of NK cell maturation is marked by the acquisition of CD57. CD56^dim^CD57^+^ NK cells are considered to be the most mature and terminally differentiated subset (32).

Contrary to this classic maturation paradigm, it is now understood that CD56^dim^ NK cells can in fact up-regulate CD56 expression upon cytokine activation and become CD56^bright^ (35). Thus, high CD56 expression does not necessarily differentiate less mature from mature NK cells, as it can also indicate mature, activated NK cells. Other recent studies provided evidence that CD57^+^ NK cells are not terminally differentiated. It was found that upon single cell expansion with K562 feeder cells, some clones derived from originally CD56^dim^CD57^+^ NK cells had lost CD57 expression. Moreover, clones from NKG2A^−^ populations were capable of reacquiring NKG2A expression (36). Following autologous stem cell transplant in patients with lymphoma or myeloma, a unique CD56^bright^ NK cell population was found to be the predominant NK cell subset following leukocyte regeneration. These young CD56^bright^ NK cells had high expression of CD57 and KIRs and potent degranulation (37). These studies demonstrate that unless NK cells are capable of regressing through maturation, classic NK cell development markers cannot definitively specify the stage of NK cell maturation.

Recent studies have also introduced discrepancies in the CD56^bright^/CD56^dim^ phenotypic dichotomization of regulatory and cytotoxic NK cells. Wagner et al. demonstrated that following priming with IL-15, CD56^bright^ pbNK cells not only had greater cytokine production compared to CD56^dim^ pbNK cells, but also displayed greater degranulation and killing in response to tumor cell targets. Importantly, they negated the possibility that this was due to up-regulation of CD56 on CD56^dim^ NK cells, as they observed this effect even with pre-sorted CD56^bright^ NK cells (38). In addition, evidence has emerged to suggest that CD56^bright^ does not unanimously define NK cells with the strongest cytokine-producing capabilities. The hepatic NK cell population is highly enriched in CD56^bright^ cells compared to pbNK cells; yet, these have recently been shown to have reduced IFN-γ and TNFα production in response to stimulation compared to pbNK cells, despite the majority of pbNK cells being CD56^dim^ (39). Building further on this phenomenon, CD56^superbright^ NK cells are considered highly immunoregulatory. Notably, CD56^superbright^CD16^−^ uterine NK cells are instrumental in promoting angiogenesis and tissue remodeling required for healthy placental development in pregnancy. These regulatory functions lend themselves as well to enhancing, rather than inhibiting, tumor progression (7). However, in stark contrast to their characteristic regulatory functions, highly cytotoxic CD56^superbright^ NK cells produced following NK cell expansion with K562-based feeder cells were recently described. These CD56^superbright^ expanded NK cells eliminated large ovarian tumors in xenograft models. What's more, within the expanded NK cell population, degranulation, cytotoxicity, and IFN-γ, increased with increasing CD56 brightness (40). Together, these studies indicate that CD56 expression cannot distinguish regulatory from cytotoxic NK cells. Indeed, CD56^bright^ NK cells can be any combination of mature or less mature, and cytotoxic or regulatory.

Since CD56^bright^ NK cells can be either cytotoxic or regulatory, the question that pursuantly arises is whether another phenotypic marker may better define these functional subsets. CD16 expression is classically used in conjunction with CD56, with CD16^+^ NK cells considered cytotoxic and CD16^−^ NK cells categorized as regulatory (13). It has been demonstrated that upon activation, CD56^dim^CD16^+^ NK cells up-regulate CD56, culminating in a CD56^bright^CD16^+^ cytotoxic NK cell population (35). Thus, it could be postulated that CD16 expression distinguishes CD56^bright^ cytotoxic and regulatory NK cells. However, highly cytotoxic IL-15-primed CD56^bright^ NK cells remained predominantly CD16^−^ (38). Moreover, Siewiera et al. have demonstrated regulatory capacities in CD16^+^ NK cells. They reported that following culture in TGF-β/IL15/IL18, pbNK cells acquired regulatory functions, as they produced high levels of VEGF, and had reduced cytotoxicity and production of IFN-γ and TNFα, but retained high expression of CD16 (41). These studies call into question the relevance of CD16 for determining cytotoxic and regulatory NK cell subsets.

**Figure 1:**
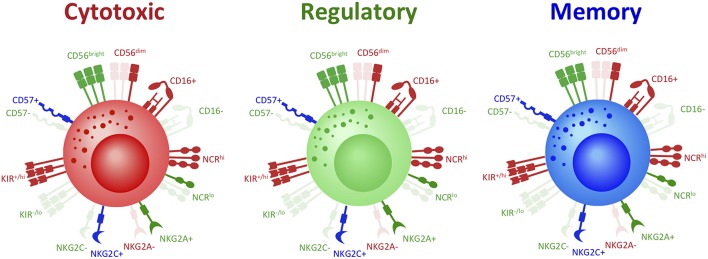
Same covers, different stories: phenotype does not distinguish NK cell functional fates. To date, phenotype has been used as a principal means of studying and classifying NK cell functional subsets. However, there is mounting evidence that demonstrates significant discrepancies in the classic phenotype definition of NK cell subsets. For instance, while cytotoxic NK cells are classically defined as CD56^dim^CD16^+^, recent studies have demonstrated that CD56^bright^ NK cells in fact hold the greatest cytotoxic potential. It has now been shown that regulatory NK cells, classically defined as CD56^bright^CD16^−^, can also in fact express CD16. Memory NK cells also present as either CD56^bright^ or CD56^dim^ and CD16^+^ or CD16^−^. Other phenotypic markers do not further distinguish NK cell subsets: cytotoxic, regulatory, and memory NK cells can all express either high, low, or absent levels of Natural Cytotoxicity Receptors (NCRs), NKG2A, NKG2C, KIRs, and CD57. Thus, not only can NK cells with the same functional fate express a range of possible phenotypes, but NK cells with opposing functions can express the same phenotype. Indeed, the range of classic NK cell phenotypic profiles are expressed across NK cell fates and are thus insufficient to distinguish NK cell functional subsets. Red denotes receptors classically associated with cytotoxic NK cells; green denotes receptors classically associated with regulatory NK cells; blue denotes receptors classically associated with memory NK cells.

Turning to other NK cell markers does not seem to address the above discrepancies. Both regulatory and cytotoxic NK cells can express high levels of activating receptors including NKG2D, NKp30, NKp44, and NKp46, but stimulation through these receptors instigates distinct functions in regulatory and cytotoxic NK cells (7, 41, 42). While it has been reported that expression of different splice variants in these receptors partly contributes to determining downstream function (41), the fundamental mechanisms that determine the receptor isoform expressed remain unknown. Inhibitory receptor expression is equally uninformative, as both regulatory and cytotoxic NK cells can express high or low levels of NKG2A and/or KIRs (32, 38, 40, 43, 44). In all, a specific combination of NK cell phenotypic markers that consistently distinguishes regulatory from cytotoxic NK cells remains elusive (Figure 1).

A similar challenge arises in phenotypically defining memory NK cells. Memory NK cells are generated in response to a number of stimuli, including hapten-induced contact hypersensitivity, infections, cytokine activation, and pregnancy (6, 45–47). Secondary responses of memory NK cells are variegated and depend on the sensitizing stimulus. Some memory NK cells have adaptive-like antigen-specific secondary responses, while others have an innate-like non-antigen-specific recall response (46, 47). What's more, memory responses have now been identified in both cytotoxic and regulatory NK cells (6, 45). However, what does remain consistent and broadly defines memory subsets is their functional capacity to have a rapid and enhanced response upon re-stimulation.

Studies have largely used phenotypic markers to define memory NK cells. Extensively studied memory NK cells include those generated in response to cytomegalovirus (CMV) infection. In humans, these have been predominantly defined as CD56^dim^NKG2C^+^ NK cells, which preferentially expand in response to acute infection. These NKG2C^+^ NK cells undergo contraction following acute infection, but persist long-term and specifically increase in response to human CMV (HCMV) re-activation and produce high levels of IFN-γ (48). Certainly, NKG2C plays a direct role in shaping HCMV adaptive NK cell responses. NKG2C on HCMV-adaptive NK cells has been shown to display fine peptide specificity through differential recognition of polymorphic UL40-encoded peptides. These UL40 polymorphisms enabled selective recognition of distinct strains of HCMV and selective activation, proliferation, and differentiation of NKG2C^+^ adaptive NK cells (49). HCMV memory NK cells are considered to have a mature phenotype, as they lack NKG2A, have lower levels of NKp30 and NKp46, and have high KIR and CD57 expression (22, 48, 50). Although NKG2C is principally used to identify HCMV-memory NK cells, recent studies have challenged the requirement for NKG2C in generating HCMV-memory NK cells. Memory NK cell responses to HCMV have been reported in NKG2C-deficient humans and upon HCMV re-activation in patients transplanted with NKG2C-deficient cord blood (51, 52). Therefore, expression of NKG2C is not necessary to define HCMV-memory NK cells.

In addition, memory NK cells do not unanimously express a classically mature phenotype. Cytokine-induced memory-like (CIML) NK cells generated by IL-12/IL-15/IL-18 pre-activation are primarily CD56^dim^, but express CD25, NKG2A, CD94, and CD69, and lack expression of KIRs and CD57 (53, 54). Memory NK cells are also not limited to the CD56^dim^ compartment. Frequencies of long-lived BCG-reactive CD56^bright^ and CD56^dim^ NK cells were shown to be elevated for up to 1 year after BCG re-vaccination in adults and were predominantly KIR2DL2/DL3^−^ and CD57^−^. BCG re-vaccination induced greater IFN-γ expression in both CD56^bright^ and CD56^dim^ NK cells for up to 1 year compared to NK cells prior to BCG re-vaccination. Furthermore, perforin expression was also enhanced upon BCG stimulation in CD56^bright^ NK cells 1 year following BCG-revaccination, compared to CD56^bright^ NK cells pre-BCG-revaccination (55). Recently, memory NK cells have been described in the context of pregnancy. Pregnancy-trained memory uterine NK cells (PTuNKs) were reported in the deciduae of multigravid women. PTuNK cells have enhanced regulatory function including VEGF production, relative to uNK cells from primigravid deciduae, which supported enhanced vascularization and tissue growth. Given their tissue-residency, it is perhaps not surprising that PTuNK cells have a unique phenotype relative to other memory subsets, as they were predominantly CD56^bright^CD16^−^, expressed high levels of NKG2C, but unchanged levels of NKp30 and NKp46 (6). It is possible that certain phenotypic traits may distinguish antigen-specific from non-specific memory NK cells. Nevertheless, there remains no unifying phenotype to identify NK cells with memory capacity (Figure 1). Furthermore, the mechanisms within NK cells that ultimately drive and identify the capacity for an enhanced secondary response are not fully elucidated.

**Figure 2:**
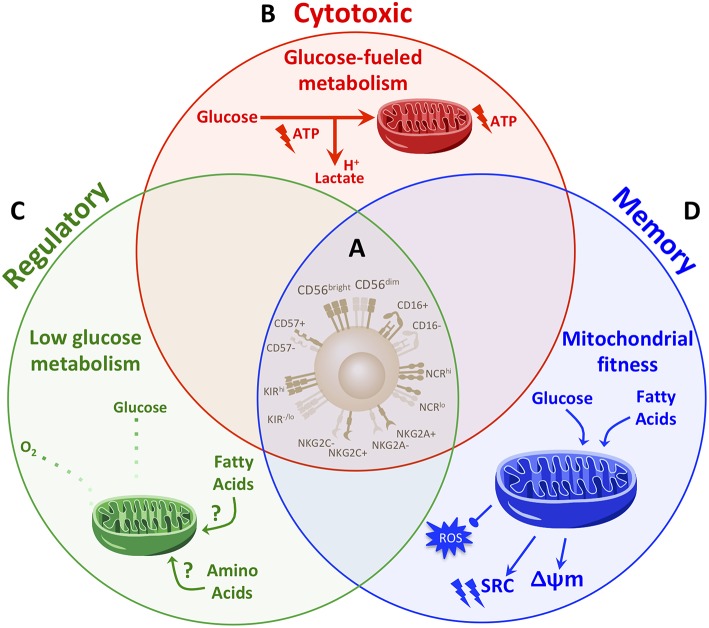
Distinct metabolic fingerprints, but not phenotype, underpin NK cell functional fates. **(A)** Though widely used to define NK cell subsets, classic NK cell phenotypic markers are proving insufficient to comprehensively identify NK cell fates. The range of archetypal NK cell phenotypes are in fact expressed across NK cells with different functional fates. In the absence of distinguishable phenotypes to reliably determine NK cell fate and functional potential, determining what drives and identifies NK cell fate will be instrumental. **(B–D)** Studies so far have demonstrated that distinct metabolic profiles drive NK cell functions. **(B)** Cytotoxic NK cells are fueled primarily by glucose. Upon activation, cytotoxic NK cells increase rates of glucose-driven glycolysis and OxPhos which in turn drive cytotoxic functions. A greater capacity for glucose metabolism through glycolysis and OxPhos identifies NK cells with the greatest cytotoxic abilities. **(C)** NK cells are polarized to a regulatory fate under hypoxic and glycolysis-limiting conditions. Thus, regulatory functions are promoted under low levels of glycolysis and OxPhos and may rely on fuels other than glucose, such as fatty acids or amino acids. **(D)** Memory NK cells exhibit enhanced mitochondrial fitness. During the contraction phase of an immune response, NK cells undergo autophagy to clear dysfunctional mitochondria, which is required for the generation of a memory NK cell pool. Memory NK cells exhibit an increased spare respiratory capacity (SRC) and membrane potential (Δψm) and reduced levels of reactive oxygen species (ROS). While memory NK cells maintain an enhanced capacity for glucose metabolism, they also up-regulate genes involved in lipid metabolism. Such a diversification in fuels in memory NK cells may provide metabolic adaptability to support longevity and the greater energy demands for enhanced function upon re-activation. Taken together, a paradigm in which distinct metabolic fingerprints comprehensively distinguish and drive the range of NK cell functional fates warrants further exploration.

If phenotype is unable to consistently identify NK cell subsets and functional fate (Figures 1, 2A), what other factors might achievably distinguish NK cell fate? Such identifying features likely lie at the heart of mechanisms that drive NK cell function.

## If an NK Cell Cannot be Defined by How It Looks, Could It be Defined by How It Is Fueled?

As highly plastic innate responders, NK cell effector fate is molded by its environment. NK cells integrate a number of environmental cues which determine downstream function. In the absence of activating signals, naïve NK cells remain in a resting quiescent state. In response to inflammatory signals, cytotoxic effector NK cell responses are marked by robust proliferation and synthesis of proinflammatory cytokines and cytotoxic machinery. Upon resolution of inflammation, NK cells curb their response, returning to a quiescent state. It is noteworthy that this shifting functional profile represents a profound shift in energy dynamics from a low-energy quiescent state to one with substantial energetic and biosynthetic requirements. Further, NK cells exert distinct functions across different tissue sites that vary greatly in nutrient types and availability. From these factors arises a parallel between NK cell energetics and functional fates.

Indeed, the capacity of a cell to generate energy through metabolism has emerged as an important factor in enabling immune cell effector functions. The burgeoning field of NK cell metabolism has uncovered the involvement of cell metabolism throughout different steps of NK cell fate determination, including development, cytotoxic effector responses, and generation of long-lived memory populations. Thus, could distinct metabolic profiles comprehensively identify and drive NK cell functional fates?

### At Their Inception, NK Cell Development Is Enabled by Metabolic Signals

NK cell development is an energy-intensive process as it requires high levels of proliferation of NK cells in the bone marrow. As NK cells progress through development, their proliferation slows and they progress to state of quiescence in the periphery (56, 57). In line with this, gene expression analysis has demonstrated that metabolic pathways associated with cell growth are up-regulated in developing NK cells whereas gene signatures associated with metabolic quiescence, such as fatty acid catabolism and aerobic metabolism, are predominant in resting NK cells in the periphery (56). While studies have yet to comprehensively measure the metabolic activity of NK cells at different stages of development, key metabolic regulators have emerged as pivotal drivers of NK cell maturation.

The mechanistic target of rapamycin (mTOR) is a major energy sensor in the cell that integrates signals for nutrient availability, growth, and activation. In response to such signals, mTOR up-regulates glycolytic metabolism and biosynthetic processes (58). A seminal study by Marçais et al. revealed the critical role for mTOR in murine NK cell development. They found that mice with an NK cell-specific deletion of mTOR had normal levels of developing NK cells in the bone marrow, but only trace levels of NK cells in the periphery (56). Other studies have further demonstrated that mTOR signaling is required for the early stages of NK cell development (19, 20). The protein E4BP4 plays an indispensible role in the commitment to the NK cell lineage by promoting the transcription of Eomes (59). Downstream of IL-15 signaling, mTOR activation by the kinase PDK1 was found to be required for E4BP4 expression in bone marrow NK cells. Indeed, knock-out of PDK1 in NK cells arrested NK cell development in early stages by inhibiting mTOR activation and reducing expression of the transferrin receptor CD71 and the amino acid transporter CD98 (20). Tight regulation of mTOR activity is critical for NK cell development, as the expression of *Tsc1*, a repressor of mTOR, is required to prevent deregulated proliferation and resulting exhaustion in response to IL-15 in developing NK cells (19). Together, these studies present mTOR-mediated metabolic signaling as a central node in NK cell development.

The evidence to date has demonstrated the requirement for metabolic regulators in NK cell development and that a shifting metabolic expression profile parallels development. These studies portray a baseline energy-intensive, biosynthetic metabolic fingerprint for developing NK cells in the bone marrow, which shifts to a more energy-conservative fingerprint as NK cells progress to the periphery. Further developing and characterizing the metabolic fingerprints of NK cells throughout development may more broadly define the maturation stages of NK cells, particularly for later stages in which phenotype fails to do so and for NK cells at different tissue sites. Assessing the baseline metabolic fingerprint in such instances holds potential to more comprehensively define the degree of NK cell maturity.

### Greater Glucose-Driven Metabolic Fitness Identifies NK Cells With the Greatest Cytotoxic Capacity

Although metabolically quiescent at baseline, mature cytotoxic NK cells up-regulate the rate of glucose-driven glycolysis and oxidative phosphorylation (OxPhos) upon stimulation (15–17, 60, 61). Increases in these metabolic pathways are accompanied by increased expression of the nutrient receptors Glut1, CD71, and CD98 (16, 56, 60). Activated cytotoxic NK cells primarily rely on glucose to fuel mitochondrial ATP production, as inhibition of glycolysis via the competitive inhibitor 2-deoxyglucose (2DG) impaired ATP production comparably to direct inhibition of OxPhos by the ATP synthase inhibitor oligomycin (61). The activation-induced increase in NK cell glucose metabolism has been shown to be regulated by mTOR, glutamine, and transcription factors cMyc and Srebp. mTOR activity is highly up-regulated in NK cells in response to stimulation (16, 56, 60, 62). In murine NK cells, knock-out or inhibition of mTOR has been shown to prevent the increases in glycolysis, glycolytic enzymes, and nutrient receptor expression upon activation (16, 56, 63). Similarly in human NK cells, the up-regulation of glycolysis in response to IL-15 and IL-2 is dependent upon mTOR, although independent from mTOR in response to IL-15/IL-12 stimulation (60). cMyc is another key driver of glucose metabolism in immune cells (64). In response to IL-2/IL-12 stimulation, glutamine uptake by NK cells was found to drive the expression of cMyc which in turn was required for the activation-induced increase in glycolysis and OxPhos by up-regulating glycolytic machinery and mitochondrial biogenesis (17). Srebp transcription factors promote the catabolism of glucose to cytosolic citrate in NK cells by supporting the activity of the citrate-malate shuttle which shuttles glucose-derived citrate from the mitochondria to the cytosol. Activity of the shuttle in turn produces mitochondrial NADH which fuels elevated OxPhos in NK cells (15).

This up-regulation in glucose-driven glycolysis and OxPhos is critical for NK cell cytotoxicity and IFN-γ production. Direct inhibition of glycolysis in murine NK cells, by 2DG or by substituting glucose with galactose in culture, inhibited IFN-γ and granzyme B expression in response various modes of stimulation including TLR-, cytokine-, or activation receptor-mediated stimulation (16, 61). In line with the evidence that glucose is the primary fuel driving OxPhos in cytotoxic NK cells, inhibition of OxPhos by glucose-depleted media or the ATP synthase inhibitor oligomycin also potently inhibited receptor-mediated IFN-γ production (61). Mah et al. demonstrated the pivotal role for NK cell glucose metabolism in the defense against infection: inhibition of glycolysis with 2DG impaired NK cell clearance of MCMV-infected cells in mice and compromised control of viremia and resulting survival of the mice (18). Arrest of NK cell glucose metabolism has also been shown to play a critical role in obesity-induced NK cell dysfunction. The increased presence of fatty acids in obesity led to lipid accumulation in NK cells, resulting in a PPAR-mediated increase in NK cell lipid metabolism and abrogation of glycolysis and OxPhos. These metabolic changes impaired polarization of cytotoxic machinery and resulting anti-tumor NK cell functions (65). Consistent with its effects on glucose metabolism, mTOR inhibition broadly impaired cytotoxic NK cell functions, including IFN-γ, perforin, and granzyme B expression and degranulation and cytotoxicity in response to tumor cell targets (56, 63). Moreover, inhibition of either cMyc expression, Srebp activity, or the citrate-malate shuttle all critically impaired NK cell IFN-γ production and cytotoxicity (15, 17). In human NK cells, elevated OxPhos is essential for NK cell cytotoxicity and IFN-γ production in response to IL-2 and IL-15/IL-12 stimulation. Heightened glycolysis was also shown to be required for NK cell cytotoxicity following 24 h IL-15 stimulation and for a robust IFN-γ response to IL-15/IL-12, particularly in CD56^bright^ NK cells (18, 60). Keating et al. demonstrated metabolic differences between CD56^bright^ and CD56^dim^ human pbNK cells (60). In line with their greater cytotoxic functions following cytokine priming, CD56^bright^ pbNK cells had enhanced mTOR-mediated expression of nutrient receptors and glucose uptake in response to cytokine stimulation as compared to CD56^dim^ pbNK cells. Thus, up-regulation of glucose-driven glycolysis and OxPhos characterizes and enables the activation of cytotoxic NK cell functions.

Accumulating evidence is demonstrating that the most highly cytotoxic NK cells are endowed with greater glucose-driven metabolic fitness. Extended pre-activation of NK cells for 3–5 days with IL-15 induces greater IFN-γ production in response to receptor-mediated activation as compared to short-term (4–24 h) IL-15 pre-activation (56, 61). During extended IL-15 stimulation, NK cells undergo profound metabolic reprogramming as compared to short-term stimulation, as they greatly increase both their rate and overall capacity for glycolysis and OxPhos (56, 61). Following such metabolic reprogramming, NK cell IFN-γ production was no longer susceptible to the inhibition of OxPhos alone; rather, aggressive inhibition of both glycolysis and OxPhos by treating NK cells with oligomycin in glucose-free media was required to suppress IFN-γ (61). Such metabolic reprogramming has demonstrated improved outcomes in the context of infection. Pre-treatment of mice with the IL-15 super-agonist complex ALT-803 for 3 days prior to MCMV infection increased basal and maximal glycolytic and oxidative metabolism and rescued impaired viral clearance in response to 2-DG treatment (18). Similar metabolic reprogramming also underpins the enhanced cytotoxic functions of licensed NK cells. The process of licensing during NK cell development requires that inhibitory KIRs expressed by the NK cell recognize self-MHC, the result of which lowers the threshold required for activation and enables greater effector potential in licensed NK cells compared to their unlicensed counterparts (66). Schafer et al. demonstrated that glycolytic metabolism is a critical regulator of NK cell licensing (21). In NK cells expanded with IL-21-expressing K562 feeder cells, highly functional licensed NK cells had greater glycolysis and glycolytic reserve compared to less functional unlicensed NK cells. Despite this increased conversion of glucose to lactate, expanded licensed NK cells sustained comparable levels of mitochondrial respiration as unlicensed NK cells, indicating a greater net level of glucose-derived energy production. While unlicensed NK cell cytotoxicity was highly sensitive to inhibition of OxPhos with oligomycin, the cytotoxicity of licensed NK cells was more resistant to metabolic inhibition. Similar to following extended IL-15 stimulation, the cytotoxicity of licensed NK cells was only inhibited upon aggressive abrogation of glucose metabolism by overnight incubation in glucose-free media, 2DG and oligomycin (21). Together, these findings demonstrate that a greater capacity for glucose-fueled energy production identifies NK cells with enhanced cytotoxic functions (Figure 2B). Moreover, greater metabolic fitness in these highly functional NK cells imparts greater flexibility in using either glycolysis or OxPhos to fuel their cytotoxic functions, rendering them more resistant to metabolic stressors.

A parallel emerges when considering the phenotype and metabolism of highly cytotoxic NK cells. It is known that upon extended cytokine stimulation including IL-15, in addition to increasing their capacity for glucose metabolism, NK cells also up-regulate CD56 expression and become predominantly CD56^bright^ (35). Indeed, evidence has separately demonstrated that 1) CD56^bright^ pbNK cells have a greater propensity for glucose metabolism compared to CD56^dim^ NK cells (67), and 2) CD56^bright^ pbNK cells have superior cytotoxicity and IFN-γ production compared to CD56^dim^ NK cells following priming with IL-15 (38). Moreover, a recent report suggests that highly functional licensed expanded NK cells have greater CD56 expression than their less functional unlicensed counterparts. Following the same feeder cell expansion with IL-21-expressing K562 cells, expanded NK cell degranulation and IFN-γ production increased with CD56 expression: the most cytotoxic and thus licensed NK cells were CD56^superbright^ whereas the least cytotoxic were CD56^dim^ (40). Together these studies expose an inextricable tie between heightened cytotoxic potential, capacity for glucose metabolism, and degree of CD56 expression. Thus, in the context of cytotoxic NK cells, both CD56 expression and heightened glucose metabolism are indicative of subsets with the greatest functional capacity. However, highly functional regulatory NK cells are also CD56^bright^, which raises the question: can distinct metabolic programs differentiate highly cytotoxic CD56^bright^ NK cells from highly regulatory CD56^bright^ NK cells?

### Glucose Metabolism—The Weight That Tips the Balance Between Cytotoxic and Regulatory NK Cells?

Research on NK cell metabolism has largely focused on the metabolic pathways that generate anti-viral and anti-tumor effector NK cells and it is clear that enhanced glucose metabolism is critical for such functions. On the other hand, while regulatory NK cells have critical homeostatic anti-inflammatory roles in the body, there is a dearth of knowledge about the role of metabolism in driving regulatory NK cell polarization and functions. In order to more fully understand the role of metabolism in the polarization of NK cell functions, it will be critical for future studies to investigate the metabolic profile of regulatory NK cells. For instance, is the metabolism of highly functional NK cells similar irrespective of whether the downstream functions are cytotoxic or regulatory? Or do regulatory NK cells predominantly utilize distinct, non-glucose-driven metabolic pathways which determine their regulatory functional fate?

The metabolism of a cell is largely dictated by its microenvironment, including factors such as nutrient and oxygen availability. Thus, in considering the above questions, it is worthwhile to examine microenvironments that favor regulatory NK cell polarization, such as the uterus, tumors, and the liver, and the metabolic profile these may shape:

Following implantation in the uterine endometrium, the early growth of the embryo occurs in hypoxic conditions in the uterus (68). Hypoxia in turn stimulates the secretion of VEGF and other angiogenic factors from uNK cells to support the demands for increased vascularization (69, 70). In addition, TGFβ plays an important role in inducing regulatory uNK cell functions (41, 69–71). Many parallels are evident between regulatory uNK cells and tumor-associated (TA-)NK cells. As a result of rapid tumor cell proliferation, tumors have aberrant vascularization resulting in large pockets of hypoxia (72, 73). In addition, high levels of anti-inflammatory cytokines such as TGFβ are produced by other tumor-associated immune cells and are known to impair NK cell anti-tumor functions (67). Similar to uterine NK cells, TA-NK cells in a number of cancers have been shown to have poor cytotoxicity, but secrete the angiogenic factors VEGF and PlGF and, through a mechanism involving TGFβ, acquire a uNK-like CD56^bright^CD16^−^ phenotype (4, 5, 74). The liver is another site enriched in regulatory NK cells and anti-inflammatory cytokines including TGFβ and IL-10 (75, 76). Liver-resident NK cells have been shown to suppress the proliferation of T cells and B cells through their secretion of IL-10, and to inhibit the anti-viral activity of T cells through PD1/PDL1 engagement (77, 78). Moreover, the liver is predominantly hypoxic, with oxygen levels dipping as low as 1.3% in the healthy liver, which can be further exacerbated by infection and fibrosis (79–82). Hypoxia has been shown to be an important factor in limiting the anti-viral activity of NK cells in HCV+ patients, but does not affect their regulatory activity against liver fibrosis (82).

Hypoxia and anti-inflammatory cytokines stand out as hallmarks across environments that foster regulatory NK cell functions. The critical role of hypoxia and TGFβ in directly polarizing NK cells to a regulatory state was highlighted by a study that demonstrated that *in vitro* culture of cytotoxic pbNK cells in TGFβ+IL-15 under hypoxic conditions was sufficient to convert pbNK cells to regulatory NK cells that secreted high levels of VEGF and had poor cytotoxicity (69). The induction of regulatory NK cells by hypoxia and TGFβ suggests that there are vastly different metabolic requirements for regulatory NK cell functions compared to the glucose-driven glycolytic and respiratory requirements for cytotoxic functions. In contrast to the ability of regulatory NK cells to thrive in hypoxic conditions and in line with the reliance of cytotoxic NK cells on glucose-driven OxPhos, hypoxia suppresses NK cell anti-tumor and anti-viral effector functions (82, 83). Studies have also demonstrated that TGFβ inhibits NK cell cytotoxic functions by suppressing glucose-driven glycolysis and OxPhos (63, 84). It follows then, that regulatory NK cell functions induced by TGFβ are not reliant on, and perhaps even inhibited by, elevated glucose metabolism. Further supporting this notion is a recent study which demonstrated that the tumor environment directly limits glycolysis in NK cells. The lung tumor microenvironment increased NK cell expression of fructose-1,6-bisphosphatase (FBP1), a rate-limiting enzyme in gluconeogenesis, which reduced glucose flux through glycolysis (85); thus, regulatory functions such as VEGF and PlGF production by TA-NK cells are unlikely to rely on elevated glucose metabolism. Another recent study demonstrated that in response to cytokine stimulation, regulatory liver-resident CD56^bright^ NK cells had lower expression of the glucose transporter Glut1, but higher expression of the amino acid transporter CD98 and the CD71 transferrin receptor compared to pbNK cells (86). These findings support a model in which regulatory NK cells are less reliant on glucose metabolism compared to cytotoxic NK cells, and may utilize other fuels, such as amino acids and/or fatty acids, to support their functions (Figure 2C).

Indeed, the evidence to date indicates that dependence on glucose-driven glycolysis and OxPhos may be a central node in tipping the balance between cytotoxic and regulatory NK cell fate. If this proves true, these distinct metabolic fingerprints would consistently distinguish cytotoxic from regulatory NK cells, addressing the drawbacks encountered in phenotypically defining these subsets. Future studies investigating the metabolic parameters that govern regulatory NK cell functions will be imperative to establishing this paradigm.

### Deep Breaths Bring Longevity—Mitochondrial Respiration at the Core of NK Cell Memory

The generation of memory NK cells requires a shift from the energy-intensive effector phase of the immune response to a contraction phase, characterized by a curtailment in proliferation and effector functions, resulting in the generation of resting long-lived memory NK cells. The ability of memory NK cells to mount a more robust and rapid effector response upon re-stimulation compared to naïve activated NK cells suggests an enhanced ability to draw upon energy reserves upon re-activation. Given the fundamental shifts between energy expenditure and quiescence between effector, contraction, and recall phases of an immune response, it is perhaps not surprising that metabolic changes have been found to be both necessary and sufficient to drive the formation and enhanced recall responses of other memory immune cells (29, 30, 87, 88).

In NK cells, restoring mitochondrial function following an effector response has been shown to be critical in the generation of memory NK cells during murine CMV infection. O'Sullivan et al. demonstrated that during the peak of the effector phase in response to MCMV, virus-specific NK cells developed reduced mitochondrial fitness, marked by a decrease in mitochondrial membrane potential and increase in ROS production. In contrast, the contraction phase was marked by the clearance of dysfunctional mitochondria in NK cells through autophagy, resulting in the restoration of mitochondrial membrane potential and reduced ROS. The clearance of dysfunctional mitochondria through autophagy was required for the generation and survival of MCMV-memory NK cells, as the inhibition of autophagy impaired the formation of the memory NK cell pool, while treatment with an ROS scavenger restored memory formation. Further, enhancement of autophagic activity through the inhibition of mTOR or activation of the metabolic regulator AMPK enriched the memory-NK cell population (23).

Enhanced mitochondrial fitness has emerged as a defining characteristic of HCMV-adaptive NK cells in humans (Figure 2D). NK cells from HCMV-seropositive donors had greater levels of maximal respiration, but not glycolysis, compared to NK cells from seronegative donors. Further, sorted CD56^dim^NKG2C^+^ HCMV-adaptive NK cells had comparable basal levels of glycolysis and OxPhos, but an increased capacity for these pathways, compared to donor-matched CD56^dim^NKG2C^−^ canonical NK cells. These findings indicate that adaptive NK cells have a greater ability to up-regulate glycolysis and OxPhos upon activation. The enhanced respiratory capacity of adaptive compared to canonical NK cells was supported by greater mitochondrial membrane potential and expression of genes involved in the electron-transport chain. The increased mitochondrial fitness of adaptive NK cells was found to be regulated by the chromatin-modifying protein ARID5B, and necessary for enhanced NK cell survival and effector function (22).

A question that remains to be addressed is which metabolic fuel(s) is/are primarily used by adaptive NK cells. The greater glycolytic capacity in HCMV-adaptive NK cells points to greater glucose metabolism. However, gene expression analysis of HCMV-adaptive NK cells also revealed an increase in genes involved in lipid catabolism (22). Interestingly, autophagy is a key regulator of lipid catabolism (89); thus, it is plausible that a diversification and flexibility in fuel sources contributes to the resilient longevity and recall effector response in HCMV-adaptive NK cells (Figure 2D).

Although the above studies have demonstrated a metabolic basis for the generation and function of memory NK cells, these have been limited to assessing adaptive NK cells in the context of CMV infection. While phenotypic variations span different memory NK cell subsets, the memory-traits of extended survival and enhanced recall effector responses, for which the metabolic adaptations are necessary in the context of CMV, are traits that remain consistent across memory NK cells. Thus it is likely that across memory NK cell subsets, increased mitochondrial fitness and fuel flexibility are broadly required to support longevity and that an enhanced respiratory capacity is necessary to derive the energy required for enhanced function upon re-activation. An important area of future study will be to verify that similar metabolic reprogramming broadly drives the formation and function of other memory-NK cell subsets, including cytokine-induced memory-like NK cells, BCG-memory NK cells, and pregnancy-trained uterine NK cells.

## Concluding Remarks

Research on NK cell biology is continuing to evolve and uncover a wider scope of NK cell functional fates. However, as knowledge on the complexity and plasticity of NK cells has grown, so too have incongruities in the classic phenotypic definitions of NK cell subsets. Classic NK cell phenotypes have undoubtedly served as an accessible measure for profiling NK cells and provide indications on NK cell activation state. Nevertheless, in many instances phenotype in fact confounds functional fates, as NK cells that express the same phenotype can have divergent functions, and NK cells with similar functional potential can express a range of phenotypic possibilities. Indeed, identifiable phenotypes that are sufficient to profile NK cell functional fates are lacking (Figure 2A). The shortcomings of phenotype in informing function expose a need to further search for what identifies NK cell fate and develop strategies to efficiently profile NK cells by these factors. Turning to a central hub that governs NK cell fate holds promise to address these drawbacks.

Metabolism has emerged as an intriguing factor to distinguish NK cell functional fates. The field of NK cell metabolism is still in early stages, but studies so far point to distinct metabolic profiles as drivers of NK cell functional potential which may thus serve as reliable fingerprints to identify functional fates (Figures 2B–D). A heightened capacity for glucose metabolism through glycolysis and OxPhos identifies NK cells with the greatest cytotoxic capacity. In contrast, regulatory NK cells thrive in hypoxic and glycolysis-limiting conditions, suggesting that regulatory functions can be effected with minimal levels of OxPhos and glycolysis. Longevity in its essence requires adaptability and endurance—memory NK cells are marked by enhanced mitochondrial fitness, achieved by the clearance of dysfunctional mitochondria, increased mitochondrial respiratory capacity and membrane potential, and reduced levels of damaging reactive oxygen species. Collectively, the evidence to date suggests a paradigm in which distinct metabolic fingerprints, but not phenotype, comprehensively distinguish and drive the range of NK cell functional fates (Figure 2).

Certainly, significant questions remain to fully delineate and validate this paradigm. For instance, what metabolic fuels and pathways are principally used by regulatory NK cells? Are low levels of glucose metabolism sufficient to support regulatory functions or do regulatory NK cells use an alternate fuel, such as fatty acids, as a more efficient means of energy generation in low oxygen conditions? Does enhanced mitochondrial fitness span all memory NK cell types? Are there metabolic pathways that further distinguish memory cytotoxic from memory regulatory NK cells? Are other factors, such as transcription markers and epigenetic modifications, required in conjunction with metabolism to determine NK cell fate? Such questions present exciting avenues for future research and will provide a more comprehensive understanding of the mechanisms and role of metabolism in tuning the spectrum of NK cell functions.

While metabolism is a promising means by which to identify NK cell fates, another critical question is whether it is achievable to efficiently profile NK cells based on metabolism. In this regard, similar to assessing classic phenotypic markers, a number of metabolic measures are frequently assessed via cytometry or microscopy, allowing for multiplex analyses that comprehensively measure a number of metabolic features. These include functional mitochondrial measures, such as mitochondrial mass, structure, membrane potential, ROS production, and oxidative stress (22, 29). Further, the relative activity of major metabolic regulators such as mTOR and AMPK are routinely measured by the phosphorylation status of these and their downstream targets (16, 56, 60, 90). Although not a direct measure of metabolic activity, the expression of cell surface nutrient receptors such as Glut1, CD71, and CD98 have been shown to reflect certain metabolic states in NK cells (16, 60). Finally, measuring the activities of key metabolic enzymes through cytometry has shown to be a valid and reliable means of assessing the metabolic configurations of immune cells and can additionally be accomplished *in situ* (91). With these applicable and efficient methods at hand, it will be imperative that future work characterize and validate combinations of metabolic measures that comprehensively indicate and discern the metabolic fingerprints of NK cell fates.

Given the critical and distinct roles that NK cells play in a host of diseases, therapeutically modulating NK cell function is garnering increasing attention. With distinct metabolic fingerprints at the heart of NK cell functional fates, profiling and modulating metabolism hold promise as powerful therapeutic strategies to predict and control NK cell fate.

## Author Contributions

SP designed and wrote the manuscript. AA designed and edited the manuscript.

### Conflict of Interest Statement

The authors declare that the research was conducted in the absence of any commercial or financial relationships that could be construed as a potential conflict of interest.
